# Gene regulatory network prediction using machine learning, deep learning, and hybrid approaches

**DOI:** 10.48130/forres-0025-0014

**Published:** 2025-07-30

**Authors:** Sai Teja Mummadi, Md Khairul Islam, Victor Busov, Hairong Wei

**Affiliations:** 1 Department of Computer Science, Michigan Technological University, Houghton, MI, USA; 2 Computational Science and Engineering, Michigan Technological University, Houghton, MI, USA; 3 College of Forest Resources and Environmental Science, Michigan Technological University, Houghton, MI, USA

**Keywords:** Machine learning, Deep learning, Transfer learning, Gene regulatory network, Convolutional neural network

## Abstract

Construction of gene regulatory networks (GRNs) is essential for elucidating the regulatory mechanisms underlying metabolic pathways, biological processes, and complex traits. In this study, we developed and evaluated machine learning, deep learning, and hybrid approaches for constructing GRNs by integrating prior knowledge and large-scale transcriptomic data from *Arabidopsis thaliana*, poplar, and maize. Among these, hybrid models that combined convolutional neural networks and machine learning consistently outperformed traditional machine learning and statistical methods, achieving over 95% accuracy on the holdout test datasets. These models not only identified a greater number of known transcription factors regulating the lignin biosynthesis pathway but also demonstrated higher precision in ranking key master regulators such as MYB46 and MYB83, as well as many upstream regulators, including members of the VND, NST, and SND families, at the top of candidate lists. To address the challenge of limited training data in non-model species, we implemented transfer learning, enabling cross-species GRN inference by applying models trained on well-characterized and data-rich species to another species with limited data. This strategy enhanced model performance and demonstrated the feasibility of knowledge transfer across species. Overall, our findings underscore the effectiveness of hybrid and transfer learning approaches in GRN prediction, offering a scalable framework for elucidating regulatory mechanisms in both model and non-model plant systems.

## Introduction

A gene regulatory network (GRN) visually represents the intricate regulatory interactions between regulators and their target genes, which collectively control metabolic pathways and biological processes essential for plant growth and development, as well as adaptation to various environmental cues and stresses^[1]^. Constructing GRNs is therefore critical for elucidating the molecular mechanisms underlying plant physiology and stress responses. With the explosion of publicly available omics data, a handful of highly efficient computational methods have been developed to infer transcription factor (TF)-target gene (TF-target) relationships. However, supervised learning approaches remain underutilized, despite their potential to leverage known regulatory interactions to accurately predict novel TF-target pairs at scale.

Although GRNs can be constructed through experimental means, for example, yeast one hybrid assay (Y1H)^[2]^, DNA electrophoretic mobility shift assay (EMSA)^[3]^, chromatin immunoprecipitation and sequencing (ChIP-seq)^[4]^, and DNA affinity purification and sequencing (DAP-seq)^[5]^, these approaches are labor intensive and low-throughput, limiting their application only to small gene sets. In contrast, *in silico* approaches based on omics data offer a scalable alternative for revealing regulatory relationships^[6−9]^. Currently, transcriptomic data sets serve as the most widely used high-throughput resource for GRN construction. The analytical strategy for transcriptomic data depends on its structure. Most publicly available datasets from plants and animals are static, often pooled from multiple non-time-course experiments such as treatment-versus-control comparisons. For such static data, suitable GRN inference methods include TIGRESS^[10]^, mutual information-based algorithms such as ARACNE^[11]^ and CLR^[12]^, and random forests-based methods such as GENIE3^[13]^, all of which infer regulatory relationships without requiring and using temporal information.

In recent years, some algorithms have been developed to construct hierarchical GRNs, such as the BWERF algorithm^[9]^, the Top-down GGM algorithm^[8]^, and the Bottom-up GGM algorithm^[7]^. Additionally, several methods are capable of constructing multiple GRNs jointly using data from multiple tissues or conditions, such as JRmGRN^[14]^ and joint graphical lasso using ADMM^[15]^. Recently, our team developed a novel method, TGPred^[16]^, which infers the target genes of each TF by integrating statistics, machine learning (ML), and optimization.

While experimental approaches and computational inference algorithms exist for GRN construction, ML, deep learning (DL), and hybrid approaches have emerged as powerful alternatives for reconstructing GRNs at scale^[17]^. Compared to experimental techniques such as yeast one-hybrid (Y1H) assays, EMSA, ChIP-seq, and DNA affinity purification sequencing (DAP-seq) − which are accurate but labor-intensive and low-throughput − ML and DL methods offer several practical advantages for genome-wide GRN prediction across diverse conditions. ML and DL models are highly scalable, enabling the analysis of large datasets where traditional experimental methods often fall short^[18]^. ML and DL approaches can capture nonlinear, hierarchical, and context-dependent regulatory relationships−features often difficult to capture with traditional statistical or rule-based methods. In particular, DL architectures such as Convolutional Neural Networks (CNNs)^[19]^ and Recurrent Neural Networks (RNNs)^[20]^ excel at learning high-order dependencies and hidden patterns in gene expression data. Tools like DeepBind^[21]^, DeeperBind^[22]^, and DeepSEA^[23]^ apply CNN-based models to predict regulatory relationships from sequence-based features. Moreover, ML and DL frameworks can integrate heterogeneous data types—including gene expression profiles, sequence motifs, and epigenetic information—to improve predictive power^[24]^. However, these benefits come with challenges. DL models typically require large, high-quality labeled datasets for effective training, which are often unavailable for many plant species. Traditional ML methods such as multiple linear regression^[25]^, Support Vector Machine (SVM)^[26]^, and Decision Trees^[27]^ can struggle with high-dimensional, noisy omics data and may fail to capture nonlinear or hierarchical relationships. Additionally, overfitting and limited interpretability can be concerns, particularly when applied to small or unbalanced datasets.

To address these limitations, various hybrid approaches that combine the feature learning capabilities of DL with the classification strength and interpretability of ML have gained traction. For example, temporal attention mechanisms integrated with LSTM architectures have been used to predict crop yield by combining genomic and environmental features^[28]^. Optimized hybrid deep learning frameworks, such as CNN-stacked LSTM architectures have been used to improve time-series prediction accuracy for environmental variables^[29]^. Similarly, MusiteDeep^[30]^ combines CNNs with attention modules to enhance kinase-specific phosphorylation site prediction. Recent work has also demonstrated that combining deep feature extraction with machine learning ensembles can improve classification performance on imbalanced datasets^[31]^. Together, these hybrid frameworks offer a flexible and robust solutions for inferring integrated regulatory networks−a broadly defined GRNs that incorporate multi-omics layers such as protein–protein interactions and metabolic pathways−especially when dealing with limited or heterogeneous datasets. By leveraging the strengths of ML, DL, and hybrid strategies, researchers gain a versatile computational toolkit for uncovering complex regulatory mechanisms, enabling large-scale, cross-context GRN construction that complements traditional experimental methods.

Transfer learning is a ML strategy that leverages knowledge acquired from one domain with large-scale datasets to improve performance in a related but less well-characterized domain with limited data^[32]^. In plant genomics and bioinformatics, transfer learning can facilitate the inference of gene regulatory relationships in a target species with limited training data by reusing models trained on a well-annotated, data-rich species. For example, Moore et al. used annotated gene expression data from *Arabidopsis thaliana* to classify specialized and general metabolism in tomato^[33]^, demonstrating the potential of transfer learning for cross-species analysis. To maximize its effectiveness, it is essential to select a source species with extensive and well-curated datasets, such as *Arabidopsis,* to support robust representation learning. Considering evolutionary relationships and the conservation of genes, especially transcription factor families, between source and target species should be considered to enhance the transferability of regulatory features. Beyond using orthologous gene expression levels and patterns, recent studies have integrated metabolic network models into transfer learning frameworks to further constrain and guide GRN reconstruction. This integration of biochemical constraints alongside transcriptomic data can significantly improve prediction accuracy by capturing the underlying biological context more effectively^[34]^.

In this study, we investigated the potential of ML, DL, and hybrid approaches for constructing GRNs using transcriptomic data from three plant species: *Arabidopsis thaliana*, poplar (*Populus trichocarpa*), and maize (*Zea mays*). Our results demonstrate that the hybrid approaches integrating ML and DL significantly outperformed traditional methods. A key challenge in GRN inference is the limited availability of experimentally validated regulatory pairs, particularly in less characterized species like poplar and maize. To address this limitation, we employed transfer learning strategies, leveraging training data of *Arabidopsis* to predict regulatory relationships in the other two species. Our results demonstrate that transfer learning, particularly when integrated with CNN-based models, significantly improves prediction performance across species. These findings underscore the promise of cross-species learning and provide a foundation for advancing knowledge transfer approaches in regulatory network inference for data-scarce plant systems.

## Methods and materials

### Data collection and preprocessing

The raw data sets in FASTQ format of *Arabidopsis thaliana*, poplar, and maize were retrieved from the Sequence Read Archive (SRA) database at the National Center for Biotechnology Information (NCBI) using the SRA-Toolkit^[35]^. The data sets were preprocessed as follows: (1) Adaptor sequences and low-quality bases were removed from raw reads using Trimmomatic (version 0.38)^[36]^; (2) Quality control was performed using FastQC^[37]^ to assess the quality of raw and processed reads. (3) The trimmed reads were aligned to each species' reference genome using STAR (2.7.3a)^[38]^, and gene-level raw read counts were obtained using CoverageBed^[39]^. These counts were subsequently normalized using the weighted trimmed mean of M-values (TMM) method from edgeR^[40]^. The normalized data sets from *Arabidopsis thaliana*, poplar, and maize were named Compendium Data Sets 1, 2, and 3, respectively. Compendium Data Set 1 includes 22,093 genes and 1,253 biological samples collected from various RNA-seq experiments (Table 1a). Compendium Data Set 2 consists of 34,699 genes and 743 biological samples, while Compendium Data Set 3 comprises 39,756 genes and 1,626 biological samples (Table 1a). The distributions of these compendium data sets from *Arabidopsis thaliana*, poplar, and maize before and after TMM normalization are shown in Supplementary Figs S1−S3, respectively.

**Table 1:** Training and testing data sets. (a) Transcriptomic compendium, training, and test data sets from *Arabidopsis thaliana*, *Populus trichocarpa*, and *Zea mays* (B73 cultivar). (b) The test datasets collected from existing databases and literature for model evaluation.

(a) Training data					
Species	Number of genes	Expression samples	Training data		
			Total	Positive pairs	Negative pairs
*Arabidopsis thaliana*	22,093 (Compendium Data Set 1)	1,253	2,462	1,231	1,231
*Populus trichocarpa*	34,699 (Compendium Data Set 2)	743	4,214	2,107	2,107
Zea mays (B73)	39,756 (Compendium Data Set 3)	1,626	16,900	8,450	8,450
(b) Test data					
Species		TFs	Targets	Expression samples	Total pairs
*Arabidopsis* Transcriptomic Test Data Set 1		1,415	20	1,253	28,300
*Arabidopsis* Transcriptomic Test Data Set 2		199	35	1,253	1,164
Poplar Transcriptomic Test Data Set		1,717	25	743	42,925
Maize Transcriptomic Test Data Set		2,555	38	1,626	97,090

### Preparation of training data sets

The Arabidopsis training data contains 1,231 pairs of experimentally validated regulatory relationships, which were obtained from the Arabidopsis Gene Regulatory Information Server (AGRIS) database^[41]^. Following that, 1,231 negative pairs were generated by randomly pairing transcription factors (TFs) with other genes in the genome, excluding the known positive regulatory pairs from AGRIS^[41]^. We then extracted the expression values of the positive and negative gene pairs from Compendium Data Set 1, resulting in Arabidopsis training dataset with 2,462 rows × 2,506 columns in the data matrix.

For poplar and maize, although a few gene regulatory relationships have been reported, most lack direct experimental validation (e.g., Y1H, ChIP-seq, DAP-seq) to confirm whether the regulatory relationships are direct. Due to the scarcity of such gold-standard data, we treated both poplar and maize as non-model species with no reliable training labels. Therefore, to enable supervised learning, we implemented a homologous gene mapping approach using the validated Arabidopsis regulatory pairs as a reference. This resulted in 2,107 putative positive gene pairs for poplar and 8,450 for maize. Negative gene pairs for both species were generated using the same strategy as for Arabidopsis—random pairing of TFs with non-target genes, excluding the homolog-based positives. Expression data for these gene pairs were then extracted from species-specific compendium datasets. For poplar, expression profiles for 2,107 positive and 2,107 negative pairs (4,214 total) were obtained from Compendium Data Set 2, yielding a matrix of 4,214 rows × 1,486 columns. For maize, 8,450 positive and 8,450 negative pairs (16,900 total) were retrieved from Compendium Data Set 3, resulting in a matrix of 16,900 rows × 3,252 columns. A summary of the training datasets for all three species is provided in Table 2.

**Table 2:** Performance comparison of fully connected networks (FCN) and convolutional neural networks (CNN) on the holdout test set (20% of the *Arabidopsis*, poplar, and maize training data). (a) FCN accuracies using binary cross-entropy (BCE), hinge loss, mean squared error (MSE), mean squared logarithmic error (MSLE), mean absolute error (MAE), Poisson loss, Huber loss, and LogCosh loss. (b) Assessment of CNNs, including custom architectures and deep CNNs (ResNet-50, MobileNet), using the same loss functions.

(a) Fully connected layer										
Species	FCN BCE	FCN HINGE	FCN MSE	FCN MSLE	FCN MAE	FCN POISSON	FCN HUBER	FCN LOGCOSH		
*Arabidopsis*	87.42	84.58	87.02	85.4	89.25	87.62	87.42	87.42		
Poplar	95.28	91.85	91.11	92.44	92.92	92.8	92.25	91.37		
Maize	89.05	88.82	89.79	89.5	89.05	90.8	90.95	90.71		
Average scores	90.58	88.42	89.31	89.11	90.41	90.41	90.21	89.83		
(b) Convolutional neural network										
Species	CNN BCE	CNN HINGE	CNN MSLE	CNN MSE	CNN MAE	CNN POISSON	CNN HUBER	CNN LOGCOSH	ResNet 50	Mobile Net
*Arabidopsis*	93.5	91.48	92.29	91.48	91.88	92.69	91.47	92.08	81.93	74.03
Poplar	97.59	98.1	97.59	97.21	98.35	97.85	97.72	96.32	88.67	85.47
Maize	94.86	95.34	90.48	94.08	95.4	88.51	95.65	94.9	85.89	83.07
Average scores	95.32	94.97	93.45	94.26	95.21	93.02	94.95	94.43	85.5	80.86

### Test data collection

To test whether the models had the potential to capture the true gene regulatory relationships, we gathered multiple test datasets in addition to the holdout test datasets (20% of positive/negative datasets in the training data sets). The test datasets include: (1) *Arabidopsis* Transcriptomic Test Data Set 1 consists of genes associated with the lignin biosynthesis pathway (LBP)^[42]^. To identify potential transcription factors regulating genes in the lignin BP, 1,415 unique TFs and 20 LBP genes were paired, resulting in 28,300 pairs for *Arabidopsis* Transcriptomic Test Data Set 1 (Table 1b). The expression values of 28,300 TF-pathway gene pairs were extracted from Compendium Data Set 1 to obtain *Arabidopsis* Transcriptomic Test Data Set 1. (2) *Arabidopsis* Transcriptomic Test Data Set 2 contains the gene pairs validated by Y1H assay reported by Taylor-Teeples et al.^[43]^. Of 623 paired TFs and their target genes, 582 pairs between 199 TFs and 35 target genes can be retrieved from Compendium Data Set 1 of Arabidopsis. This data set comprises 582 regulatory relationships between 199 TFs and 35 target genes validated by using Y1H. A corresponding negative testing data set of 582 gene pairs was generated as described above. The 582 positive regulatory pairs were combined with 582 randomly generated negative pairs, resulting in a total of 1,164 regulatory pairs. After extracting their expression values from Compendium Data Set 1 (Table 1b), the *Arabidopsis* Transcriptomic Test Data Set 2, containing 1,164 rows × 2,506 columns, was generated (Table 1b). (3) Poplar Transcriptomic Test Data Set was prepared using 25 LBP genes and 1,717 TFs of poplar. Pairwise combination of 25 LBP genes with 1,717 TFs resulted in a total of 42,925 regulatory pairs. After extracting their expression values from Compendium Data Set 2 (Table 1a), we obtained the Poplar Transcriptomic Test Data Set, which contains 42,925 rows and 1,486 columns (Table 1b). (4) Finally, the maize Transcriptomic Test Data Set was prepared by pairing 38 LBP genes with 2,555 TFs as described above, resulting in a test data set with 97,090 rows and 3,252 columns (Table 1b).

### Machine learning

Multiple ML methods were used to analyze the gene expression data. More specifically, supervised models, a category of ML techniques, require each data point to have a specific label. Supervised ML models, such as Support Vector Machines (SVM), Decision Trees (DT), Logistic Regression (LR), and K-Nearest Neighbors (KNN), have demonstrated strong performance in inferring GRNs^[26,44−46]^. For example, Gillani et al.^[26]^ demonstrated the effectiveness of SVMs in predicting gene regulatory relationships, while Parry et al.^[45]^ highlighted the predictive power of KNN models in gene expression analysis. Ensemble learning methods such as Random Forest (RF), Extremely Randomized Trees, AdaBoost Models, Gradient Boosting, have demonstrated promising results in GRN prediction^[47]^. Additionally, bagging^[48]^ was applied as an ensemble technique to further enhance the robustness and accuracy of the GRN predictions.

Hyperparameter tuning is a crucial step in constructing effective ML models since each model has specific adjustable parameters that depend on the data type and distribution. The hyperparameters are selected based on their impact on each model's performance and their ability to mitigate overfitting or underfitting. The grid search technique was employed to select hyperparameters by testing various combinations on the models.

### Deep learning

Deep learning approaches, particularly convolutional neural networks (CNNs), have demonstrated strong performance in modeling complex gene expression patterns^[20]^ and regulatory relationships^[49,50]^. CNNs are a class of neural network architectures inspired by biological neurons and designed to capture spatial or structural patterns in data. In this study, neural networks were implemented using the Keras library with TensorFlow as the backend^[51]^. The construction of a customized fully connected network (FCN) for gene expression data requires configuring multiple hidden layers, each with its own set of training-specific hyperparameters, such as neuron count, activation function, learning rate, and batch size. Previous research has demonstrated that incorporating multiple layers with dropout structures can enhance FCN performance^[52]^. Based on preliminary tuning, our neural network architecture included two dense (fully connected) layers with 256 and 128 neurons, respectively, each followed by a dropout layer. An optimized learning rate of 0.00003 was determined through experimentation and used consistently across the FCN, CNN, and hybrid models. All models were trained with a batch size of 100 using the RMSprop optimizer.

### Hybrid architecture

**Figure 1:**
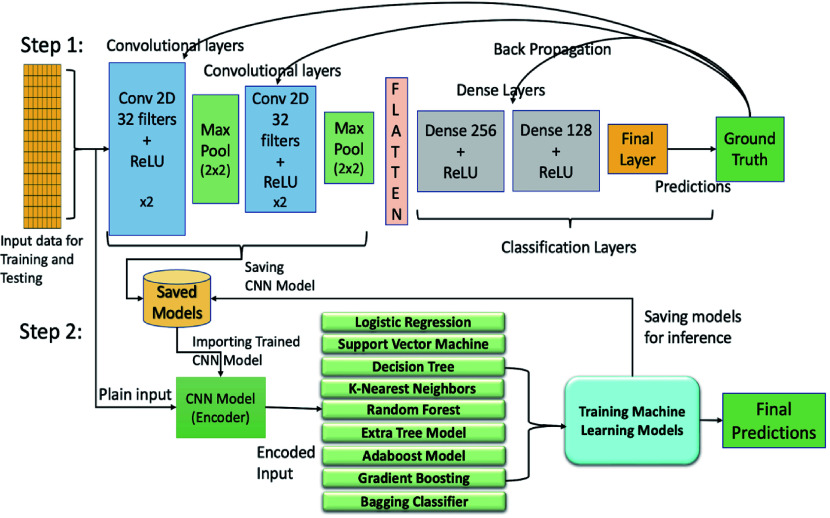
Illustration of the architectures of hybrid models combining machine learning (ML) and deep learning (DL) approaches. Step 1 includes training of the convolutional neural networks (CNN) using back propagation while Step 2 uses the outputs from the convolutional encoder/feature extractor of Step 1 to train ML models. Conv, convolutional layer; ReLU, rectified linear unit.

Hybrid architecture combines neural networks and ML algorithms, leveraging the strengths of both for effective gene expression data classification^[53]^. Our hybrid architecture consists of two steps: the feature extractor, also known as the convolutional encoder, and the classification model. In Step 1, the feature extractor*,* or convolutional encoder*,* plays a critical role in the hybrid architecture. As illustrated in Fig. 1, the input data is first passed through a series of convolutional and max-pooling layers, followed by the dense layers for training and classification. The convolutional layers act as feature extractors, while the dense layers serve as the classifier. The outputs from the convolutional layers are flattened before being passed to the dense layers. Once training is complete, the convolutional model and its learned weights are stored. In Step 2, for classification, the outputs from the feature extractor are used to train ML models. Supervised ML models such as LR, SVM, DT, and KNN, as well as ensemble techniques like RF, Extremely Randomized Trees, AdaBoost, and Gradient Boosting, are trained on the extracted features and stored for inference and performance evaluation.

### Performance metrics

This section discusses the performance metrics used to evaluate and compare model performance on the test data. These metrics include accuracy, precision, recall, specificity, F1 score, and the Receiver Operating Characteristic (ROC) curve. AdaBoost, and Gradient Boosting, are trained on the extracted features and stored for inference and performance evaluation. The accuracy metric gives the percentage of correct predictions made by the model in relation to the total number of predictions made by a model.

$ \begin{aligned}\rm Accuracy=\;&\dfrac{\mathrm{N}\mathrm{u}\mathrm{m}\mathrm{b}\mathrm{e}\mathrm{r}\;\mathrm{o}\mathrm{f}\;\mathrm{c}\mathrm{o}\mathrm{r}\mathrm{r}\mathrm{e}\mathrm{c}\mathrm{t}\;\mathrm{p}\mathrm{r}\mathrm{e}\mathrm{d}\mathrm{i}\mathrm{c}\mathrm{t}\mathrm{i}\mathrm{o}\mathrm{n}\mathrm{s}}{\mathrm{T}\mathrm{o}\mathrm{t}\mathrm{a}\mathrm{l}\;\mathrm{n}\mathrm{u}\mathrm{m}\mathrm{b}\mathrm{e}\mathrm{r}\;\mathrm{o}\mathrm{f}\;\mathrm{p}\mathrm{r}\mathrm{e}\mathrm{d}\mathrm{i}\mathrm{c}\mathrm{t}\mathrm{i}\mathrm{o}\mathrm{n}\mathrm{s}}\\=\;&\dfrac{\mathrm{T}\mathrm{r}\mathrm{u}\mathrm{e}\;\mathrm{P}\mathrm{o}\mathrm{s}\mathrm{i}\mathrm{t}\mathrm{i}\mathrm{v}\mathrm{e}\;\left(\mathrm{T}\mathrm{P}\right)\mathrm{ }+\mathrm{ }\mathrm{T}\mathrm{r}\mathrm{u}\mathrm{e}\;\mathrm{N}\mathrm{e}\mathrm{g}\mathrm{a}\mathrm{t}\mathrm{i}\mathrm{v}\mathrm{e}\mathrm{s}\;\left(\mathrm{T}\mathrm{N}\right)}{\mathrm{T}\mathrm{o}\mathrm{t}\mathrm{a}\mathrm{l}\;\mathrm{n}\mathrm{u}\mathrm{m}\mathrm{b}\mathrm{e}\mathrm{r}\;\mathrm{o}\mathrm{f}\;\mathrm{s}\mathrm{a}\mathrm{m}\mathrm{p}\mathrm{l}\mathrm{e}\mathrm{s}}\end{aligned} $

Precision is the ratio of the true positive predictions made by the algorithm out of all the positive predictions. Also, a high precision value indicates that the algorithm has generated very few false positives.

${\mathrm{ Precision}} = \rm\dfrac{True\;Positive\;\left(TP\right)}{True\;Positive\;\left(TP\right) +False\;Positive\;\left(FP\right)} $

Recall is the ratio of the true positive predictions to the sum of true positives and false negatives. Recall is also known as sensitivity.

$ {\mathrm{Recall}} = \rm\dfrac{True\;Positive\;\left(TP\right)}{True\;Positive\;\left(TP\right) +False\;Negative\;\left(FN\right)} $

Specificity is a measurement that tells us how many of the negative predictions are correctly made. Specificity is the ratio of the true negative predictions to the total number of negative instances in the dataset.

$ {\mathrm{Specificity }}= \rm\dfrac{True\;Negative\;\left(TN\right)}{True\;Negatives\;\left(TN\right) +False\;Positives\;\left(FP\right)} $

F1-score is the harmonic mean of the precision and recall values, ranging from 0 to 1. A higher F1-score indicates a good balance between precision and recall, whereas a score near 0 suggests poor model performance and a significant imbalance between these two metrics.

$ \mathrm{F1\; Score}=2\ \times\ \dfrac{Precision\ \times\ Recall}{Precision+Recall} $

ROC curve is a performance measurement curve for the classification problems. The ROC curves are plotted as true positive rate (TPR; y-axis) vs false positive rate (FPR; x-axis), where FPR is 1 - specificity. The points on the ROC curve are plotted at different threshold levels based on the probabilities of the prediction. AUC, the area under a ROC curve, represents the model's ability to distinguish between classes and is a measure of overall classification performance; a higher AUC indicates better separability between positive and negative classes.

## Results

Multiple ML GRN prediction models with distinct architectures were assessed, including four supervised learning models—LR, SVM, DT, KNN, and five ensemble learning models—RF, Extremely Randomized Trees, AdaBoost Models, Gradient Boosting, and Bagging Classifier. Additionally, neural network approaches including FCNs and CNNs with various loss functions and architectures, were examined. Hybrid models combining ML and CNN were also implemented and evaluated. We present training results and model evaluations across multiple test datasets. Initially, the outcomes of hyperparameter tuning for the ML models are discussed, followed by an analysis of cross-validation scores and the accuracy of the models on holdout test data. These models were trained separately for different species and evaluated using their respective holdout test data sets. Moreover, a brief overview of the training and tuning for FCNs, CNNs, and hybrid models is provided, including their accuracies on the holdout test data. In addition to using the holdout test data, the models were also assessed with the real test data sets: *Arabidopsis* Transcriptomic Test Data Set 1, *Arabidopsis* Transcriptomic Test Data Set 2, Poplar Transcriptomic Test Data Set, and Maize Transcriptomic Test Data Set.

### Hyperparameter tuning and testing ML models

In this study, *Arabidopsis* Training Data was used for hyperparameter tuning with the grid search technique. For LR, we conducted a grid search across multiple parameters, ultimately selecting values that ensured stable convergence while maintaining model simplicity. Our tests confirmed that the L2 penalty type provided optimal regularization without eliminating features, while the 'saga' solver demonstrated superior efficiency regardless of dataset size and effectively handled the regularization term. In the case of SVM, our grid search explored various kernel functions to determine the optimal decision boundary configuration. Through systematic testing, we identified how different kernel parameters affected the non-linearity of the decision boundary and selected the most effective combination for our specific dataset. The KNN algorithm underwent extensive parameter tuning, where we compared different distance metrics. The Manhattan distance metric emerged as more robust for our feature space compared to Euclidean distance. Through grid search, we identified the optimal number of neighbors that achieved the best balance between noise reduction and preservation of local decision boundaries. For the models like DT, RF, Extremely Randomized Trees, our grid search focused on parameters controlling model complexity to prevent overfitting. We systematically tested various maximum depth values to find the optimal point where models maintained sufficient expressiveness without becoming too specific to the training data. Similarly, we tuned the minimum samples for leaf and split nodes through grid search, carefully considering how these parameters affected the inherent randomness in feature selection. For ensemble techniques like Adaboost, Gradient boosting, our grid search explored different combinations of estimators and learning rates. The results showed that selecting an appropriate number of estimators coupled with a small learning rate allowed the models to learn gradually and effectively reduced the risk of overfitting. Various hyperparameters tuned and learned for different ML models applied on the *Arabidopsis* Training Data are shown in Supplementary Table S1. The tuned hyperparameters were applied to the ML models for further training and prediction.

**Figure 2:**
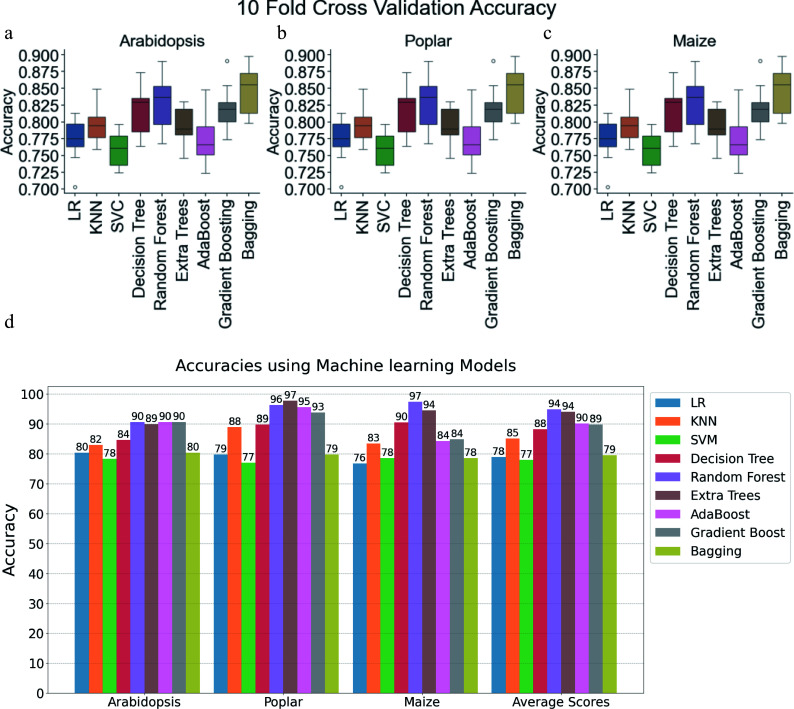
Performance evaluation of machine learning models and convolutional neural networks on *Arabidopsis*, poplar, and maize datasets. Boxplots showing 10-fold cross-validation accuracies for Logistic Regression, K-Nearest Neighbors (KNN), Support Vector Classifier (SVC), Decision Tree, Random Forest, Extremely Randomized Trees (ExtraTrees), AdaBoost, Gradient Boosting, and Bagging on training data for (a) *Arabidopsis*, (b) poplar, and (c) maize. (d) Clustered bar chart comparing model accuracies within and across species using holdout test data (20% of training data). Each bar corresponds to a specific model's performance. LR: Logistic Regression, SVM: Support Vector Machine, KNN: K-Nearest Neighbor.

After tuning the hyperparameters, 10-fold cross-validation was applied to evaluate and compare the performance of ML models across three species: *Arabidopsis*, poplar, and maize. Figure 2a−c displays the classification accuracies of several models, including LR, KNN, SVM, and DT, as well as several ensemble models such as RF, Extremely Randomized Trees, AdaBoost Model, and Gradient Boosting, and Bagging. Each figure corresponds to the model performance using training data from *Arabidopsis*, Poplar, and Maize Training Data, respectively. The boxplots in Fig. 2a−c show the first quartile, median, and third quartile of the accuracy values. Based on the median accuracy scores, the ensemble models RF, Gradient Boosting, and Bagging performed better than the other methods. After hyperparameter tuning, the ML models were trained on 80% of the *Arabidopsis*, Poplar, and Maize Training Data (as shown in Table 1a), and tested with 20% reserved as the holdout test data. The results of these evaluations are presented in Fig. 2d.

Several models, including LR, SVM, DT, and KNN exhibited relatively lower average accuracies, ranging from 76% to 90% across all three plant species compared to other models. Ensemble techniques, such as Random Forest, Extremely Randomized Trees, AdaBoost Classifier, and Gradient Boosting, outperformed all other models on the 20% holdout test data. AdaBoost and Gradient Boosting achieved average accuracies of approximately 90%. Random Forest and Extremely Randomized Trees demonstrated the best performance, with average accuracies exceeding 94% on the holdout test data.

### Hyperparameter tuning and testing neural networks

The FCNs were trained using 80% of the training data of *Arabidopsis*, poplar, and maize with different loss functions, including BCE, hinge loss, MSE, MSLE, MAE, Poisson loss, Huber loss, and LogCosh loss. These were tested separately on the 20% holdout test data. The results are shown in Table 2a. The FCN with the binary cross entropy (BCE) loss had an average accuracy of 90.58%, which indicates good performance on the holdout test data of *Arabidopsis*, poplar, and maize training data.

**Figure 3:**
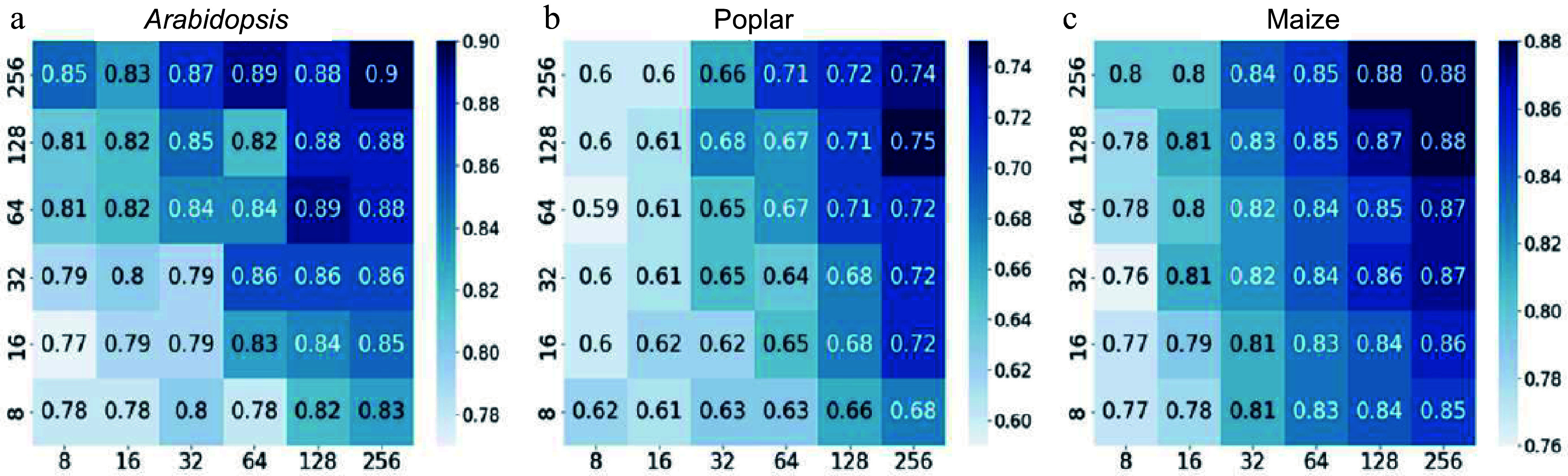
Heatmaps depicting accuracy values of convolutional neural networks (CNN) with varying numbers of kernels in the first and second layers. The y-axis represents the number of kernels in the first layer of the CNN, whereas the x-axis represents the number of kernels in the second layer. Binary cross-entropy was used as the loss function for the CNN, and the performance was evaluated on a 20% holdout test set of the *Arabidopsis*, poplar, and maize training data. The scale bars represent a series of accuracy values. (a) CNN accuracy on *Arabidopsis* holdout data. (b) CNN accuracy on poplar holdout data. (c) CNN accuracy on maize holdout data.

The next step was to conduct hyperparameter tuning of the CNN to identify optimal training parameters. It is essential to choose the appropriate number of layers and kernels to construct an effective, customized CNN model. To assess which kernel combinations were suitable for the gene expression data, two stacked convolutional layers were used, and different numbers of kernels (8, 16, 32, 64, 128, 256) were sequentially applied with a kernel size of 3 × 3. BCE was used as the loss function to train and evaluate these models. Figure 3a−c display the heatmaps of the accuracy values. As a result, multiple kernel numbers were tested for the three species under investigation.

The results revealed that CNN performance with BCE loss improved on *Arabidopsis* data when increasing kernel numbers in the first and second layers (Fig. 3a). However, this enhancement was relatively less evident in poplar and maize species, with 256 kernels in *Arabidopsis* yielding 90% accuracy, while only 74% in poplar 88% in maize (Fig. 3b & c). These findings indicate that increasing kernel counts does not universally lead to better performance across species. To further improve model accuracy, we systematically explored architectural modifications, including stacking convolutional layers with max pooling, and varying the structure of dense layers and dropout rates. Instead of repeating the same configuration across models, we compared multiple combinations of layer sizes, dropout values, and optimizers. The final CNN architecture−selected based on its superior performance−was the result of these iterative optimization steps and aligned with the general configuration as described earlier.

Based on the resulting accuracies for the holdout test data presented in Table 2b, the CNN model with the BCE loss function achieved an average of 95.32% accuracy and outperformed other models. Other loss functions, such as MAE, Hinge Loss, and Huber loss, displayed similar performance, with average accuracy values of 95.21%, 94.97% and 94.95%, respectively, across the three species. In addition to custom-built CNNs, two deep CNNs such as ResNet^[54]^ and MobileNet^[55]^ were also used to predict regulatory relationships. The accuracies of these models on the 20% holdout test data in three species are also shown in Table 2b.

### Training and testing hybrid models

**Figure 4:**
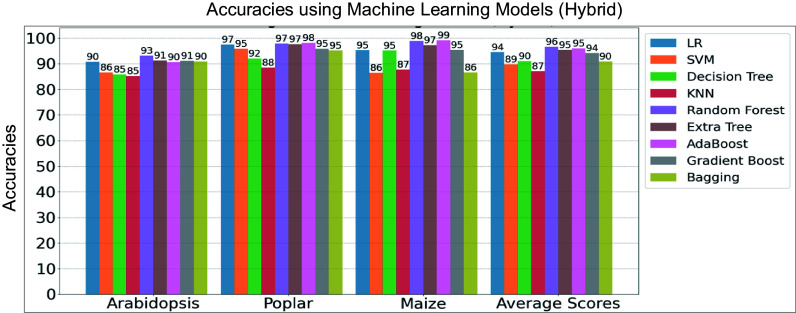
Performance comparison of hybrid machine learning (ML) and deep learning (DL) models on the holdout test data sets of three species. The y-axis denotes model accuracies for *Arabidopsis*, poplar, and maize, including average scores while the x-axis lists the species, including average scores of three species. LR, Logistic Regression, SVM, Support Vector Machine, and KNN, K-Nearest Neighbors.

As shown in Fig. 1, the training of the hybrid architecture involves two stages. In the first stage, the convolutional encoder models were trained separately on the *Arabidopsis*, poplar, and maize training datasets for 100 epochs using the Binary Cross-Entropy (BCE) loss function. Each dataset was split into 80% for training and 20% for testing. Training and testing accuracy and loss curves for these convolutional neural networks are shown in Supplementary Figs S4 and 5. These encoders served as feature extractors for the next stage. In the second stage, the extracted features were used to train various ML models on the same species-specific training data. Figure 4 shows the hybrid model accuracy on their respective holdout test data sets.

Among the ensemble learning methods, the hybrid Random Forest, hybrid Extremely Randomized Trees, and hybrid AdaBoost models excelled, achieving average accuracy scores of 96.45%, 95.42%, and 95.46%, respectively, across the three species (Fig. 4). Notably, within the hybrid architectures, Logistic Regression and Gradient Boosting models also outperformed other classifiers such as Support Vector Machine, Decision Tree, K-Nearest Neighbors, and the Bagging Classifier, based on average accuracies. The holdout test results confirmed that the hybrid Random Forest Model was the top performer across both regular and hybrid approaches, followed by Extremely Randomized Trees and AdaBoost models in the hybrid architectures. In the neural networks, BCE loss exhibited better performance on the holdout test data compared to the other loss functions. To further evaluate the effectiveness of these models, we utilized real test data consisting of genes involved in LBP as described below.

#### Testing hybrid models using Arabidopsis Transcriptomic Test Data Set 1

*Arabidopsis* Transcriptomic Test Data Set 1 consists of genes involved in LBP. The top 50 TFs predicted by the hybrid and plain machine learning models were extracted and shown in Table 3. The frequency values of each TF were calculated within the top 1,000 predicted regulatory pairs for both the hybrid and plain Random Forest Models, reflecting the strength with which each TF impacts LBP. TFs shown in red font are the known regulators of the LBP based on published literature, such as MYB7^[56]^ and WRKY12^[57]^. TFs in red fonts and yellow highlight are the known master regulators of the LBP, including MYB83 and MYB46^[58]^. TFs in blue fonts act upstream of *MYB83* and *MYB46* in the regulatory hierarchy. These include NST1, SND1/NST3^[59]^, VND1^[60]^, VND6^[61]^, and VND7^[62]^, all of which influence the expression of *MYB83* and *MYB46*. Further upstream, E2FC^[63]^ and MYB26^[64]^ acts as high-order regulators of *VND1*, *6* and 7, *SND1*, *NST1,* and *NST3* genes.

**Figure 5:**
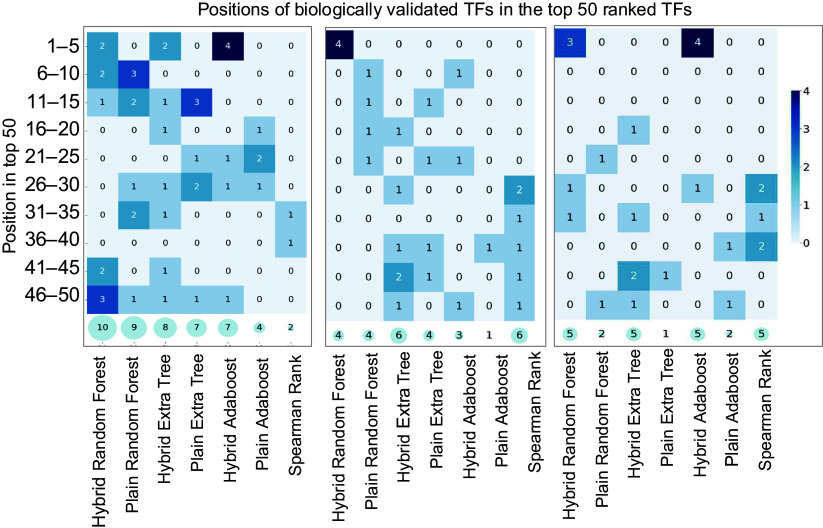
Heatmaps illustrating model performance on *Arabidopsis*, Poplar, and Maize Transcriptomic Test Datasets. The x-axis lists models, while the y-axis groups correct predictions within ranked intervals (e.g., 1–5, 6–10, …, 45–50). Darker scheme color indicates higher accuracy, and semi-transparent circles at the bottom of each plot display the total correct predictions in the top 50 genes.

The hybrid Random Forest Model successfully identified 10 known true TFs, while the plain Random Forest Model detected nine from *Arabidopsis* Transcriptomic Test Data Set 1. As a baseline comparison, we included Spearman's rank correlation coefficient − a widely used statistical measure in gene expression analysis that captures both linear and non-linear monotonic relationships^[65]^, for comparison. The Spearman's rank correlation identified two (Table 3). These results demonstrate that the hybrid model not only outperformed the plain Random Forest model in identifying known TFs but also surpassed the performance of the correlation-based method. To further illustrate the prioritization capability of each method, we collapsed the list of known *Arabidopsis* LBP regulators identified in Table 3 and visualized their ranks among the top 50 TFs in Fig. 5a. The hybrid model not only recovered more known TFs but also consistently ranked them higher among the top predictions, underscoring its superior ability to prioritize biologically meaningful candidates.

**Table 3:** Comparison of the top 50 transcription factors (TFs) predicted to regulate the lignin biosynthesis pathway (LBP) by three methods: hybrid Random Forest, plain Random Forest, and a baseline method using Spearman's rank correlation.

Hybrid Random Forest Model					Plain Random Forest Model					Spearman Correlation Coefficient			
Rank	TF	Freq.	Ref.		Rank	TF	Freq.	Ref.		Rank	Transcription Factor	Freq.	Ref.
1	AT3G08500_MYB83	20	[58]		1	AT4G36920_AP2	20	−		1	AT5G60100	6	−
2	AT1G71930_VND7	20	[62]		2	AT5G16560_KAN	20	−		2	AT4G13640	6	−
3	AT4G36920_AP2	20	−		3	AT2G20180_bHLH15	20	−		3	AT3G50700	6	−
4	AT2G20180_bHLH15	20	−		4	AT5G11260_HY5	20	−		4	AT1G64530	6	−
5	AT5G11260_HY5	20	−		5	AT2G44730	20	−		5	AT1G20693	6	−
6	AT5G16560_KAN	20	−		6	AT1G71930_VND7	17	[62]		6	AT1G04250	6	−
7	AT1G24260_SEP3	20	−		7	AT3G08500_MYB83	17	[58]		7	AT5G37020_ARF8	5	−
8	AT1G32770_SND1	19	[59]		8	AT5G12870_MYB46	16	[58]		8	AT4G31060	5	−
9	AT1G14350_FLP	19	−		9	AT1G66140_ZFP4	14	−		9	AT3G58680_MBF1B	5	−
10	AT5G12870_MYB46	18	[58]		10	AT1G24260_SEP3	12	−		10	AT3G23210	5	−
11	AT2G02820_MYB88	18	−		11	AT3G13890_MYB26	12	[64]		11	AT3G21175	5	−
12	AT4G23810_WRKY53	16	−		12	AT2G02820_MYB88	11	−		12	AT2G34710_HB-14	5	−
13	AT3G27920_GL1	14	−		13	AT1G14350_FLP	11	−		13	AT2G01650	5	−
14	AT5G62380_VND6	10	[61]		14	AT1G32770_SND1	10	[59]		14	AT1G71692	5	−
15	AT1G24625_ZFP7	10	−		15	AT5G17300	8	−		15	AT1G67970	5	−
16	AT1G74930_ORA47	9	−		16	AT2G32370	6	−		16	AT1G49720_ABF1	5	−
17	AT2G43010_AtbHLH9	8	−		17	AT1G25340_MYB116	6	−		17	AT1G19270	5	−
18	AT5G13790_AGL15	7	−		18	AT1G25330_bHLH75	6	−		18	AT5G63280	4	−
19	AT3G24650_ABI3	6	−		19	AT4G27330	6	−		19	AT5G53200_TRY	4	−
20	AT4G18960_AG	6	−		20	AT2G18060	6	−		20	AT5G46910	4	−
21	AT3G02310_AGL4	6	−		21	AT2G40220_ABI4	6	−		21	AT5G41920_GRAS-28	4	−
22	AT1G69120_AP1	6	−		22	AT1G23420_INO	6	−		22	AT5G13080	4	−
23	AT1G26310_CAL	6	−		23	AT4G35700	6	−		23	AT4G34610	4	−
24	AT2G44730	6	−		24	AT5G18450	6	−		24	AT4G17900	4	−
25	AT3G54340_AP3	5	−		25	AT2G44745	6	−		25	AT4G00050_bHLH16	4	−
26	AT1G69180_CRC	5	−		26	AT4G00220_LBD30	6	−		26	AT3G54620	4	−
27	AT5G10120_EIL4	5	−		27	AT3G27920_GL1	6	−		27	AT3G17609	4	−
28	AT1G23420_INO	5	−		28	AT1G09540_MYB61	6	−		28	AT3G16280	4	−
29	AT1G01060_LHY	5	−		29	AT2G44745_WRKY12	6	[57]		29	AT3G02830_ZFN1	4	−
30	AT5G57520_ZFP2	5	−		30	AT4G00220	6	−		30	AT2G43000	4	−
31	AT2G40220_ABI4	4	−		31	AT2G18060_VND1	6	[60]		31	AT2G40740_WRKY55	4	−
32	AT5G15800_AGL2	4	−		32	AT1G09540	6	−		32	AT2G37630_MYB91	4	−
33	AT2G45650_AGL6	4	−		33	AT1G12610	6	−		33	AT2G16720_MYB7	4	[56]
34	AT2G16910_AMS	4	−		34	AT5G62380_VND6	6	[61]		34	AT1G70000	4	−
35	AT1G25340_MYB116	4	−		35	AT3G06120_bHLH45	6	−		35	AT1G22070	4	−
36	AT1G12610_DDF1	4	−		36	AT4G09960_STK	5	−		36	AT1G17460_TRFL3	4	−
37	AT1G47870_E2FC	4	[63]		37	AT3G30530	5	−		37	AT1G12260_VND4	4	[60]
38	AT3G13960_GRF5	4	−		38	AT3G01530	5	−		38	AT1G04550	4	−
39	AT2G33880_HB3	4	−		39	AT1G61110	5	−		39	AT5G67480	3	−
40	AT5G62020_HSF6	4	−		40	AT2G42830_SHP2	5	−		40	AT5G66630	3	−
41	AT1G67100_LOB40	4	−		41	AT5G03790_LMI1	5	−		41	AT5G65410_hb-25	3	−
42	AT2G46770_NST1	4	[59]		42	AT1G15360_SHINE1	5	−		42	AT5G63080	3	−
43	AT5G20240_PI	4	−		43	AT1G66380_MYB114	5	−		43	AT5G61380_TOC1	3	−
44	AT4G27330_SPL	4	−		44	AT1G35490	5	−		44	AT5G60120	3	−
45	AT2G44745_WRKY12	4	[57]		45	AT5G23260_AGL32	5	−		45	AT5G58010	3	−
46	AT1G10480_ZFP5	4	−		46	AT2G46770_NST1	5	[59]		46	AT5G57620	3	−
47	AT2G45420_LBD18	3	−		47	AT5G15800_AGL2	5	−		47	AT5G56860_GNC	3	−
48	AT3G13890_MYB26	3	[64]		48	AT1G26310	5	−		48	AT5G54230	3	−
49	AT5G57620_MYB36	3	−		49	AT4G18960_AG	5	−		49	AT4G00180_YAB3	3	−
50	AT2G18060_VND1	3	[60]		50	AT5G53210_bHLH98	5	−		50	AT1G10200_WLIM1	3	−
The ranking is based on the frequency with which each TF is predicted to regulate genes in the lignin biosynthesis pathway (BLP). TFs shown in red font present the known regulators of LBP, based on published literature. TFs in red font and yellow highlight are the known master regulators of LBP (e.g., MYB83 and MYB46). TFs in blue font act further upstream in the regulatory hierarchy, directly or indirectly influencing the expression of *MYB83* and *MYB46*^[66]^.													

#### Testing hybrid models using Arabidopsis Transcriptomic Test Data Set 2

**Figure 6:**
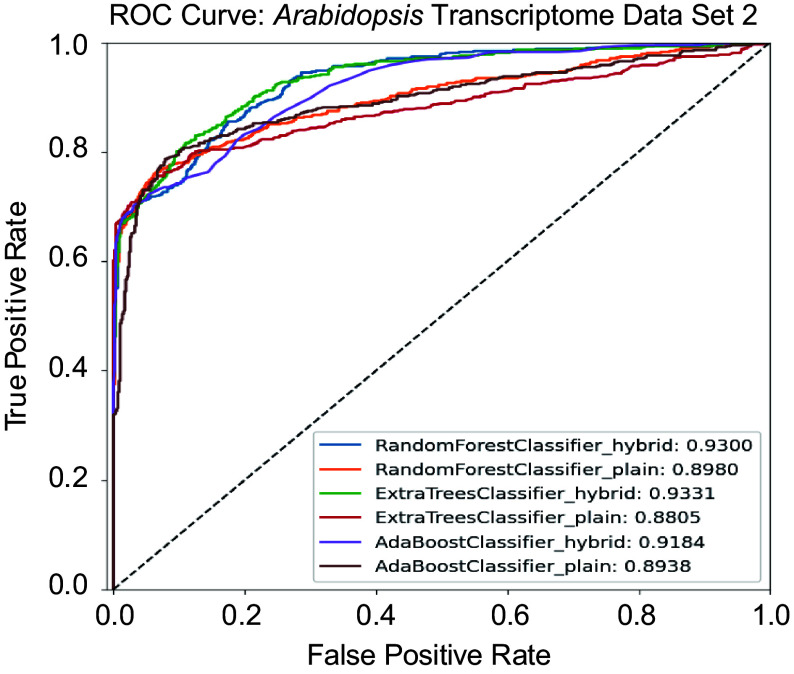
Receiver operating characteristic (ROC) curves comparing the performance of hybrid and plain models on the *Arabidopsis* Transcriptomic Test Data Set 2.

*Arabidopsis* Transcriptomic Test Data Set 2, adopted from Taylor-Teeples et al.^[43]^, contains 582 Y1H experimentally validated true regulatory relationships. We then applied Random Forest, Extremely Randomized Trees, and AdaBoost models using both hybrid architectures and conventional machine learning methods to predict regulatory relationships. The hybrid Random Forest Model identified 471 pairs of Y1H-validated true relationships. In comparison, the hybrid Extremely Randomized Trees and hybrid AdaBoost models identified 443 and 425 true relationships, respectively. The plain Random Forest model correctly identified 420 positive regulatory pairs, while the plain Extremely Randomized Trees and plain AdaBoost models detected 435 and 464 true relationships, respectively. We also compared these ML/DL methods to traditional methods, for example, Spearman rank correlation^[65]^, GENIE3^[13]^, and CLR^[12]^. Spearman rank correlation identified 403 positive relationships, while GENIE3, which ranked first in the DREAM5 network inference challenge, identified 440 positive relationships. Another top player of DREAM5 competition, CLR, which uses mutual information and contextual Z-score, identified 241 positive pairs. These findings demonstrate that hybrid models of ML and DL enhance GRN prediction accuracy compared to their plain counterparts and other traditional inference methods such as GENIE3 and CLR. Table 4 presents the performance metrics—accuracy, precision, recall, specificity, F1-score, and area under the curve (AUC) score for each model. The receiver ROC curves for all models were plotted in Fig. 6. The results shown in Table 4 revealed that tree-based models, including Random Forest and Extremely Randomized Trees, demonstrated superior performance in the hybrid architectures with AUC scores of 93.00% and 93.31%, respectively. Moreover, hybrid models consistently outperformed their plain ML counterparts. Although the hybrid models achieved higher AUC scores compared to their plain counterparts, they exhibited slightly lower values in threshold-dependent metrics such as accuracy, precision, and specificity. This discrepancy is not uncommon, as AUC reflects a model's overall ability to rank positive and negative instances across all thresholds, whereas accuracy and precision are evaluated at a fixed threshold. The higher AUC indicates better general discriminatory power, suggesting that hybrid models more effectively separate true regulatory relationships from non-regulatory ones, even if their performance at a specific classification threshold is modestly lower.

**Table 4:** Accuracy, precision, recall, specificity, F1-score, and area under the curve (AUC) score for *Arabidopsis* Transcriptomic Test Data Set 2. The data set contains 1,164 regulatory pairs, with 582 positive regulatory pairs and 582 negative regulatory pairs.

No.	Model	Accuracy	Precision	Recall	Specificity	F1-score	AUC score
1	Random Forest Classifier Hybrid	83.26	83.33	83.26	85.59	83.25	93.00
2	Random Forest Classifier Plain	84.55	86.19	84.54	95.20	84.37	89.80
3	Extra Trees Classifier Hybrid	85.15	85.38	85.15	89.19	85.12	93.31
4	Extra Trees Classifier Plain	84.03	85.60	84.03	94.51	83.85	88.05
5	AdaBoost Classifier Hybrid	81.20	81.33	81.20	84.39	81.18	91.84
6	AdaBoost Classifier Plain	84.98	85.23	84.97	89.19	84.95	89.38

Finally, we assessed the enrichment of transcription factor binding sites (TFBSs) in the predicted target genes from *Arabidopsis* Transcriptomic Test Data Set 2. Using a motif locator program^[67]^, we searched for TF-specific motifs within the promoter regions of candidate target genes. Among the 582 validated regulatory relationships, there are 199 distinct TFs and 44 unique target genes. Position weight matrix (PWM) information was available for 141 of the TFs. The hybrid Random Forest model—the top-performing approach—predicted 471 positive TF–target pairs. Of these, 339 pairs involved TFs with available PWM data. The motif locator program confirmed the presence of corresponding binding motifs in 200 of these 339 predicted regulatory pairs, providing additional support for the accuracy of the model's predictions.

#### Testing hybrid models using the Poplar Transcriptomic Test Data Set and the Maize Transcriptomic Test Data Set

After hybrid and plain models were trained and fine-tuned using the poplar and maize training data, we performed feature importance analysis using three hybrid models: the hybrid Random Forest, the hybrid Extremely Randomized Trees, and the hybrid AdaBoost models on the Poplar and Maize Transcriptomic Test Data Sets. For comparison, we also included their corresponding plain models and Spearman's rank correlation method. In this study, we highlighted two representative results: the hybrid Extremely Randomized Trees model on the poplar test set and the hybrid AdaBoost model on the Maize test set.

The results of the hybrid Extremely Randomized Trees model on poplar are shown in Supplementary Table S2. The hybrid Extremely Randomized Trees model identified seven TFs that directly or indirectly regulate LBP, including two master regulators, Potri.009G061500_MYB83 and Potri.001G267300 _MYB83, of LBP, and four further upstream regulators, Potri.019G083600_VND7, Potri.013G113100_VND7, Potri.017G016700_SND2, Potri.007G135300_SND2, and Potri.002G023400_E2FC^[63]^. The plain Extremely Randomized Trees identified five of these seven TFs identified by hybrid models but missed two *SND2* genes. In contrast, the Spearman's rank correlation method identified two SND2, Potri.017G016700_SND2 and Potri.007G135300_SND2 identified by the hybrid Extremely Randomized Trees model, plus four different regulators, Potri.007G014400_VND2, Potri.017G119900_C3H14^[68]^, Potri.004G095100_C3H14^[68]^, and Potri.001G112200_KNAT7^[69]^. C3H4 was reported to up-regulate CCOAMT and CAD6 and down-regulate LAC17, while KNAT7 functions below MYB46 and MYB83 and targets multiple LBP genes^[70]^. These results demonstrate that the hybrid Extremely Randomized Trees model outperformed the plain Extremely Randomized Trees model by identifying a greater number of known lignin-related TFs than the plain model and Spearman's rank correlation method.

Another example of feature importance analysis we conducted is a hybrid AdaBoost Model on Maize Transcriptomic Test Data Set. As presented in Supplementary Table S3. The hybrid AdaBoost model identified five known LBP regulators: Zm00001eb076470_VND7, Zm00001eb176840_VND7, Zm00001eb093920_MYB46, and Zm00001eb410950_MYB46. In contrast, the plain AdaBoost model identified two known LBP TFs, Zm00001eb076470_VND7 and Zm00001eb139600_AtMYB73. Spearman's rank correlation identified five known LBP TFs: Zm00001eb403720_KNAT7, Zm00001eb001720_KNAT7, Zm00001eb157260_SND2, Zm00001eb260850_NST2, and Zm00001eb269810_NST2. Both the hybrid AdaBoost model and Spearman's rank method identified five known LBP regulators; however, the hybrid model ranked them at the very top of the list, while the Spearman method ranked them near the bottom, demonstrating the hybrid model's superior ability to prioritize known regulators.

### CNN-based cross-species transfer learning and model performance

**Figure 7:**
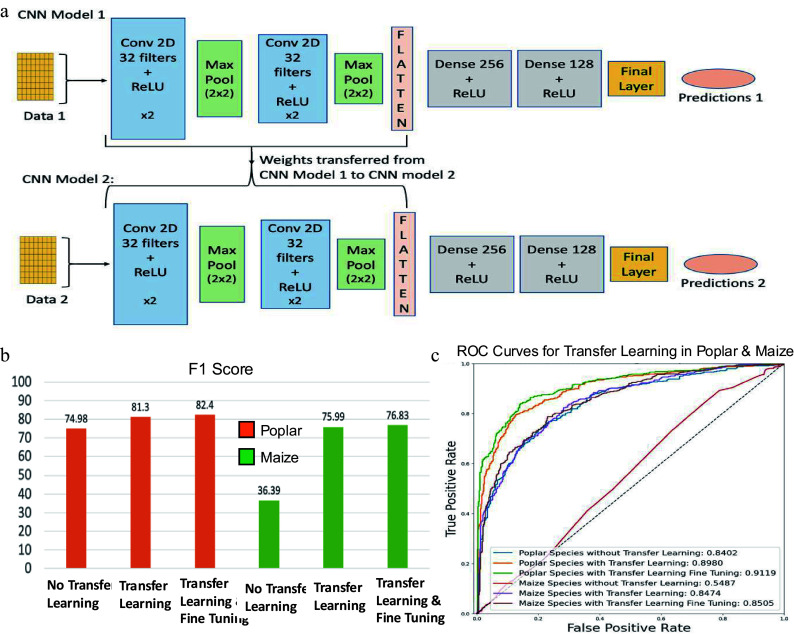
Transfer learning framework using convolutional neural networks (CNN) and its performance evaluation. (a) Overview of CNN-based transfer learning architecture. CNN Model 1 is trained on a well-characterized species (e.g., *Arabidopsis*), and its learned convolutional layer parameters are transferred to CNN Model 2, which is fine-tuned on a smaller dataset from a less-characterized species (e.g., Poplar or Maize). Conv: Convolutional layer; ReLU: Rectified Linear Unit. (b) F1-scores assessing the performance of CNN models for poplar and maize, with and without transfer learning and fine-tuning. (c) Receiver operating characteristic (ROC) curves assessing the performance of CNN models for poplar and maize, with and without transfer learning and fine-tuning.

In this study, we implemented a transfer learning approach to enable knowledge transfer from *Arabidopsis* to poplar and maize. This strategy allowed model training on poplar and maize using fewer samples than were required for *Arabidopsis*. The architecture of the transfer learning framework based on CNNs is illustrated in Fig. 7a. Model 1, referred to as the base CNN model, was trained using 80% *Arabidopsis* training data for 100 epochs, utilizing Binary Cross-Entropy (BCE) as the loss function, the RMSprop optimizer with a learning rate of 0.00003, and a batch size of 100. Once the model was successfully trained, its performance was evaluated on the 20% holdout test data of *Arabidopsis*. CNN Model 1 demonstrated an accuracy of 95.95% and an AUC score of 96.03% for *Arabidopsis* holdout test data. After Model 1 was successfully trained and tested with *Arabidopsis* training and testing data, the learned parameters for the convolutional layers were transferred to CNN Model 2 (Fig. 7a). CNN Model 2 was then fine-tuned separately using poplar and maize training data and tested using their respective testing data sets. For poplar and maize, the training datasets each included 100 positive and 100 negative regulatory pairs (Table 1a), while the test sets contained 500 positive and 500 negative pairs per species (Table 1b). CNN Model 2 was trained in two stages: first, without fine-tuning, where the transferred weights were frozen and only the final layers were trained on the species-specific data; second, with fine-tuning, where the entire model, including the transferred weights, was further trained on the species-specific data. For the poplar species, CNN Model 2 demonstrated an F1-score of 74.98% (Fig. 7b) and an AUC score of 84.02% without transfer learning (Fig. 7c). However, after incorporating the weights trained on *Arabidopsis* with CNN Model 1, the F1-score significantly increased to 81.3%, and the AUC score rose to 89.80%. Fine-tuning of these models led to further improvements in performance, yielding an F1-score of 82.4% and an AUC score of 91.19%. For the maize species, without transfer learning, the model's F1-score was only 36.39% and an AUC score of 54.87%. In contrast, after incorporating the weights trained from Arabidopsis's CNN Model 1, the transfer learning model achieved an F1-Score of 75.99% and an AUC score of 84.74%. Fine-tuning the model further enhanced its performance to an F1-score of 76.83% and an AUC score of 85.05%.

For both poplar and maize, transfer learning significantly improved model accuracy and AUC scores (Fig. 7c), with further fine-tuning leading to additional performance gains. These findings underscore the value of leveraging prior knowledge from one species to another, as it enhances the model's generalization and predictive accuracy. Consequently, transfer learning and fine-tuning are powerful tools for enhancing the performance of CNN models in plant classification and have potential applications across various other domains and tasks.

## Discussion

Gene regulatory networks (GRNs) play a central role in elucidating the regulatory mechanisms that govern metabolic pathways, biological processes, and complex traits in plants. However, GRN construction remains a major challenge due to the intricate nature of regulatory interactions and the limitations of experimental methods, which are often labor-intensive, time-consuming, and difficult to scale. In this study, we addressed these challenges using supervised learning approaches that integrate prior biological knowledge with gene expression data. Specifically, we employed a combination of machine learning (ML), deep learning (DL), and hybrid methods to predict regulatory relationships from large-scale transcriptomic datasets. To further improve performance and generalizability, we implemented transfer learning strategies that leverage regulatory knowledge from well-characterized species and apply it to less-studied plant systems. This integrated computational framework enables scalable, cross-species GRN inference and offers a practical foundation for identifying and prioritizing candidate regulators for downstream experimental validation.

Our research demonstrates the effectiveness of ML, DL, and hybrid techniques for predicting GRNs by leveraging prior knowledge and transcriptomic data from multiple plant species, including *Arabidopsis thaliana*, poplar, and maize. We systematically evaluated several ML and DL architectures − including FCNs and CNNs − across a range of loss functions. Among the FCN models tested with eight different loss functions, the model using BCE achieved the highest average accuracy (90.58%). Similarly, among CNN models tested with ten loss functions, the CNN with BCE loss achieved the best performance, reaching an average accuracy of 95.32%, followed by models using mean absolute error (MAE), hinge loss, and Huber loss (Table 2). Building on these results, we developed hybrid models that integrate CNN-based feature representations with traditional ML classifiers, using BCE loss for consistency. The hybrid Random Forest, hybrid Extremely Randomized Trees, and hybrid AdaBoost models showed substantial improvements over their non-hybrid counterparts, consistently outperforming them on both holdout and independent test datasets. These findings highlight the value of combining DL-driven feature extraction with ML-based classification for accurate, scalable, and cross-species GRN inference.

Using *Arabidopsis* Transcriptomic Test Data Set 1, and Poplar and Maize Transcriptomic Test Data Sets, we evaluated the performance of three hybrid Random Forest, hybrid Extremely Randomized Trees, and hybrid AdaBoost—against their corresponding plain versions in identifying known positive TFs regulating LBP. Spearman's rank correlation was also included as a baseline statistical method for comparison. We revealed the following facts: (1) The hybrid models not only identified a greater number of TFs but also consistently ranked them among the top candidates, highlighting their superior ability to prioritize biologically relevant regulators. (2) Notably, the hybrid models consistently identified the master regulators of LBP, MYB46 and MYB83, which were rarely detected by their plain counterparts and Spearman's rank correlation. (3) In addition, the hybrid models demonstrated greater competency in identifying upstream regulators of LBP—such as VND6/7/1, NST1/3, SND1/2, E2FC, and MYB26—compared to the plain models, while Spearman's rank method identified these regulators only sporadically.

Our analysis using *Arabidopsis* Transcriptomic Test Data Set 2 demonstrates that hybrid ML and DL models outperformed both plain ML models and traditional inference methods, including GENIE3, CLR, and Spearman's rank correlation, in predicting experimentally validated regulatory relationships. Among the evaluated models, tree-based hybrid approaches achieved the highest AUC scores (Fig. 6, Table 4), indicating superior capability in distinguishing true regulatory interactions. Although these models exhibited slightly lower accuracy and precision at fixed classification thresholds, their stronger overall discriminatory power highlights the advantages of hybrid architectures in GRN inference. Furthermore, motif enrichment analysis confirmed the biological relevance of the predicted regulatory pairs: a substantial proportion of the predicted target genes contained TF binding sites in their promoter regions. Collectively, these results underscore the improved predictive performance and biological interpretability offered by hybrid ML/DL frameworks for reconstructing gene regulatory networks.

A key limitation of our study is the limited availability of high-quality training datasets for all three species—*Arabidopsis*, poplar, and maize. While the *Arabidopsis* training set was constructed using experimentally validated regulatory pairs from the AGRIS database, equivalent datasets are largely unavailable for poplar and maize. Although some gene regulatory relationships have been reported in the literature for poplar and maize, they often lack solid experimental evidence. For example, upregulation or downregulation of a gene of interest by a regulator does not necessarily imply there is a direct regulation between them, and high-throughput methods such as Y1H, ChIP-seq, and DAP-seq are known to yield substantial noise and false positives and need to be substantiated by other experimental means. Careful curation is essential to collect, classify, and validate these data before they can be reliably used for ML or DL model training. Consequently, we treated poplar and maize as non-model species with no reliable prior knowledge of gene regulatory interactions − reflecting a realistic, data-scarce scenario. To enable supervised learning under these constraints, we generated training data by applying a homologous gene mapping strategy, using validated regulatory pairs from *Arabidopsis*. This cross-species gene mapping approach allowed us to leverage existing knowledge where direct evidence was lacking. However, it relies on the assumption that certain regulatory relationships and modules are conserved across species—an assumption that may not always hold over large evolutionary distances. Despite this limitation, the strategy enabled preliminary modeling and prioritization of candidate regulatory interactions in non-model species. Importantly, our results suggest that this approach is still practical: we were able to identify key pathway regulators in poplar and maize, albeit in smaller numbers and with lower predictive efficiency compared to *Arabidopsis*. These findings highlight both the promise and the limitations of knowledge transfer in cross-species GRN inference.

Constructing GRNs using ML and DL approaches requires robust and diverse training data. However, the amount of data available for each species in public repositories remains insufficient to support analyses specific to tissues, conditions, or developmental stages. As a result, we compiled gene expression matrices from a variety of tissues, conditions, and developmental stages to expand the available training data. While this inclusive strategy facilitates the development of generalizable models, it may obscure regulatory relationships specific to some tissues, conditions, or developmental stages. We focused on the LBP, which is active across multiple tissue types and therefore suitable for generalized modeling. However, to enable the future construction of tissue-, condition-, or stage-specific networks, it is essential to expand public databases and curate existing gene regulatory knowledge. Concurrently, transfer learning provides an alternative practical approach for extending regulatory knowledge from *Arabidopsis*—a well-characterized model species—to less-studied species such as poplar and maize. By pretraining a convolutional encoder on *Arabidopsis* data and subsequently fine-tuning it with transcriptomic data from poplar and maize, we achieved significant improvements in prediction accuracy, reduced training time, and enhanced cross-species applicability. These findings underscore the complementary roles of intra-species data integration and cross-species transfer learning in improving GRN inference under real-world data limitations.

Although limited by data availability, our study demonstrates the strong potential of machine learning (ML), deep learning (DL), and hybrid models for gene regulatory network (GRN) inference. Future research should refine hybrid approaches by, for example, incorporating time-series data and applying temporal models—such as recurrent neural networks (RNNs) or one-dimensional convolutional neural networks (1D CNNs)—to better capture dynamic regulatory processes that static models often miss. In addition, using lightweight CNNs, more advanced ML frameworks, and interpretability tools such as SHAP^[71]^ could further enhance efficiency, model performance, and transparency. Moreover, our recent findings support the exploration of attention-based architectures to construct context-dependent GRNs and identify key regulators, as recently demonstrated in Islam et al.^[72]^. Furthermore, integrating multi-omics data, including chromatin accessibility and epigenomic profiles, may further improve prediction accuracy. Given the complexity of ML/DL-based GRN models, the development of explainable artificial intelligence (AI) frameworks will be essential to improve interpretability and facilitate biological insights. Finally, transfer learning strategies should be further refined to enhance cross-species prediction. In addition to relying on orthologous gene mappings, emerging species-agnostic approaches can learn shared patterns in data structures—for example, aligning patterns of expression or co-expression modules across species, even when direct gene orthology is unavailable^[73]^. To support these advancements, we emphasize the importance of broader data sharing and the public deposition of high-resolution, tissue-specific datasets and experimentally validated regulatory interactions.

## Conclusions

This study demonstrated the effectiveness of integrating machine learning (ML) and convolutional neural network (CNN) approaches for constructing gene regulatory networks (GRNs) in plants. Using publicly available transcriptomic data and known regulatory relationships from *Arabidopsis thaliana*, poplar, and maize, we trained ML, DL, and hybrid models. The hybrid models, which combined CNN and ML techniques, outperformed their plain ML counterparts and traditional methods such as Spearman's rank correlation, GENIE3, and CLR. These models not only identified a greater number of known TFs involved in lignin biosynthesis but also consistently ranked them among the top predictions, demonstrating superior prioritization capability. Notably, the hybrid models reliably detected the master regulators MYB46 and MYB83, which were rarely identified by plain models, and showed improved ability to uncover upstream regulators such as VND6, VND7, VND1, NST1, NST3, SND1, SND2, E2FC, and MYB26. Although CNN performance was limited by the size of training datasets, transfer learning significantly improved prediction accuracy and enabled effective cross-species inference. These findings highlight the value of hybrid architectures and transfer learning in enhancing GRN prediction and offer a scalable framework for translating regulatory knowledge across species. This integrative approach advances our capacity to explore gene regulatory mechanisms underlying metabolic processes, developmental programs, and complex traits in both model and non-model plant systems.

## SUPPLEMENTARY DATA

Supplementary data to this article can be found online.

## Data Availability

The python code is available at https://github.com/SaiTejaMummadi/Gene_Regulatory_Analysis or http://prosper.ffr.mtu.edu/.
