# Supplementary material for: Gene regulatory network prediction using machine learning, deep learning, and hybrid approaches
Source: For Res (Fayettev). 2025 Jul 30;5:e014. doi: 10.48130/forres-0025-0014 (PMC12441907; doi:10.48130/forres-0025-0014)
Supplement: Supplementary file 1 — Supplementary data to this article can be found online. [file FR-2025-5-0014-Supplementary.zip › 10.48130_forres-0025-0014-Suppl-FigureS4.pdf]

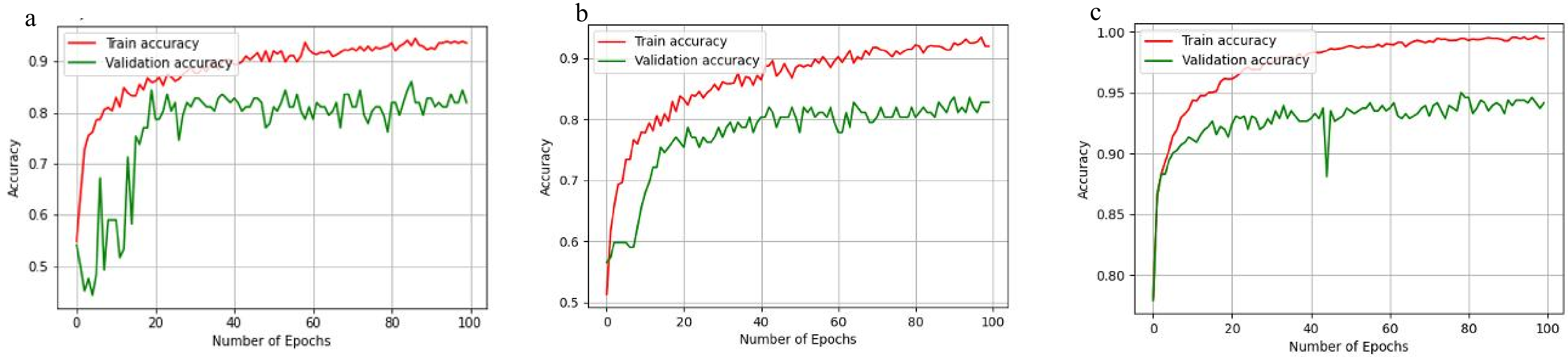

Supplementary Fig. S4. Training and validation accuracy curves of the convolutional neural networks shown in the step 1 of the Hybrid architecture.

Figure (a), (b), and (c) represents the accuracies of the *Arabidopsis thaliana*, poplar (*Populus trichocarpa*) and maize (*Zea mays*) training data.
