# Supplementary material for: Gene regulatory network prediction using machine learning, deep learning, and hybrid approaches
Source: For Res (Fayettev). 2025 Jul 30;5:e014. doi: 10.48130/forres-0025-0014 (PMC12441907; doi:10.48130/forres-0025-0014)
Supplement: Supplementary file 1 — Supplementary data to this article can be found online. [file FR-2025-5-0014-Supplementary.zip › 10.48130_forres-0025-0014-Suppl-TableS1.pdf]

Supplementary Table S1: The hyperparameters learned through training and the description for different models using *Arabidopsis* Training Data

Model	Parameter tuned	Description
Logistic Regression	penalty: l2, C: 1, solver: saga, max iterations: 500	**Penalty** is a regularization parameter; **C** is the inverse of regularization strength; **solver** is the optimization algorithm; **max iterations** define the number of iterations the algorithm performs on data
Support Vector Machine	C: 0.5, kernel: linear, gamma: scale, degree: 4	**C** is the regularization parameter; **kernel** function is used to map data to higher dimensions; **gamma** controls the shape of the decision boundary; **degree** defines degree of the polynomial
Decision Tree	criterion: gini, min samples leaf: 5, min samples split: 5	**Criterion** is used to measure the quality of the split; **min samples leaf** is the minimal number of samples in a leaf. **min samples split** is the minimum number of samples that are required to split a node in tree.
K-Nearest Neighbors	metric: Manhattan, n neighbors: 6	**Metric** is used to calculate the distance between datapoints; **n neighbor** defines the number of neighbors considered for prediction
Random Forest	maximum depth: 20, min samples leaf: 5, min samples split: 2, estimators: 50	**Maximum depth** is the depth of each Decision Tree, **min samples leaf** is the minimal number of samples in a leaf and **min samples split** is the minimum number of samples required to split a node in tree, **estimators** are the number of Decision Trees constructed
Extremely Randomized Trees	maximum depth: 20, min samples leaf: 1, min samples split: 2, estimators: 50	**Maximum depth** is the depth of each Decision Tree, **min samples leaf** is the minimal number of samples in a leaf and **min samples split** is the minimum number of samples required to split a node in tree; **estimators** are the number of Decision Trees constructed
AdaBoost	estimators: 100, learning rate: 0.1	**Estimators** value is the number of Decision Trees constructed; **learning rate** controls the contribution of the weak classifier in final ensemble
Gradient Boosting	estimators: 100, learning rate: 0.1	**Estimators** value is the number of Decision Trees constructed; **learning rate** controls the contribution of the weak classifier in final ensemble
Bagging Classifier	estimators: 100, max samples: 10, max features: 1	Estimators value is the number of Decision Trees constructed; **max samples** define the maximum samples to use for each copy; **max features** define the maximum features to use for each copy of the model
