# Supplementary material for: Gene regulatory network prediction using machine learning, deep learning, and hybrid approaches
Source: For Res (Fayettev). 2025 Jul 30;5:e014. doi: 10.48130/forres-0025-0014 (PMC12441907; doi:10.48130/forres-0025-0014)
Supplement: Supplementary file 1 — Supplementary data to this article can be found online. [file FR-2025-5-0014-Supplementary.zip › 10.48130_forres-0025-0014-Suppl-TableS2.pdf]

Supplementary Table S2. Comparison of the Top 50 transcription factors (TFs) predicted to regulate the lignin biosynthesis pathway by Hybrid Extremely Randomized Trees and Plain Hybrid Extremely Randomized Trees on Poplar Transcriptomic Test Data Set. The frequency of each TF within the top 2000 predicted regulatory relationships was calculated to represent how many pathway genes it might have inferred. Red font indicates TFs that are known regulators of the lignin biosynthesis pathway, based on published literature. Red font with yellow highlight indicates TFs that are recognized as master regulators of lignin biosynthesis (e.g., MYB83, MYB46). Blue font denotes TFs that act further upstream in the regulatory hierarchy, influencing the expression of MYB83 and MYB46.

Hybrid Extremely Randomized Trees Model				Plain Extremely Randomized Trees Model				Spearman Correlation Coefficient			
Rank	Transcription Factor	Freq.	Reference	Rank	Transcription Factor	Freq.	Reference	Rank	Transcription Factor	Freq.	Reference
1	Potri.007G046200_AP2	24	-	1	Potri.002G252800_bHLH15	24	-	1	Potri.013G039100_At5g28300	14	-
2	Potri.002G168700_WRKY53	24	-	2	Potri.006G251800_HY5	24		2	Potri.018G129800_YABBY5	14	-
3	Potri.001G088600_ARF9	24	-	3	Potri.005G140700_AP2	24	-	3	Potri.003G036900_At1g55110	13	-
4	Potri.002G139500_At2g44730	24	-	4	Potri.018G029500_HY5	23		4	Potri.001G137800_At5g46880	13	-
5	Potri.003G169600_SEP3	24	-	5	Potri.008G210900	23	-	5	Potri.014G152000_At1g05230	13	-
6	Potri.002G172800_ARF9	24	-	6	Potri.007G046200_AP2	22	-	6	Potri.004G020400	13	-
7	Potri.011G075800_AG	24	-	7	Potri.017G137600_KAN	21	-	7	Potri.004G230800	13	-
8	Potri.001G092900_WRKY53	24	-	8	Potri.004G082400_KAN	21	-	8	Potri.019G045900	13	-
9	Potri.005G140700_AP2	24	-	9	Potri.002G172800_ARF9	21	-	9	Potri.014G107200_At3g61970	12	-
10	Potri.003G138600_WRKY53	24	-	10	Potri.002G168700_WRKY53	20	-	10	Potri.011G083100	12	-
11	Potri.017G137600_KAN	24	-	11	**Potri.001G267300_MYB83**	19	Zhong et al., 2012	11	Potri.015G075600_GL1	12	-
12	Potri.014G096200_WRKY53	24	-	12	Potri.017G107500	19	-	12	Potri.014G099900_At1g01250	12	-
13	Potri.004G082400_KAN	24	-	13	Potri.001G327100_TCP20	19	-	13	Potri.014G037200_KAN4	12	-
14	Potri.006G251800_HY5	24		14	Potri.019G123500_WRKY33	18	-	14	Potri.015G104200_bHLH137	12	-
15	Potri.002G252800_bHLH15	24	-	15	Potri.004G108320	16	-	15	Potri.015G022000_TRY	12	-
16	Potri.018G029500_HY5	24		16	Potri.014G096200_WRKY53	16	-	16	Potri.003G046700	12	-
17	Potri.001G058400_SEP3	24	-	17	Potri.008G148400_FLP	16	-	17	Potri.013G054000_NAC	12	-
18	**Potri.001G267300_MYB83**	23	Zhong et al., 2012	18	Potri.003G138600_WRKY53	15	-	18	Potri.003G169100	12	-
19	Potri.008G148400_FLP	23	-	19	Potri.006G221800_TT2	15	-	19	Potri.012G104900_bHLH137	12	-
20	Potri.009G055700_AGL15	23	-	20	**Potri.002G023400_E2FC**	15	Han et al, 2022	20	Potri.016G136500	11	-
21	Potri.002G164400_WRKY22	23	-	21	**Potri.009G061500_MYB83**	15	Zhong et al., 2012	21	Potri.005G205400_At2g43000	11	-
22	Potri.004G064300_AG	23	-	22	Potri.013G153400_WRKY33	15	-	22	Potri.002G041700	11	-
23	Potri.014G051200_At2g44730	22	-	23	Potri.014G100100_ARF9	15	-	23	Potri.005G246700	11	-
24	Potri.013G153400_WRKY33	22	-	24	Potri.010G093000_FLP	14	-	24	Potri.017G094800_TFPD	11	-
25	Potri.014G090300_WRKY22	22	-	25	Potri.002G139500_At2g4473	14	-	25	Potri.004G050150_ARF3	10	-
26	**Potri.002G023400_E2FC**	21	Han et al, 2022	26	Potri.011G007800_WRKY42	14	-	26	Potri.010G223300_GATA-8	10	-
27	Potri.017G082900_hb-34	21	-	27	Potri.014G090300_WRKY22	14	-	27	**Potri.007G014400_VND2**	10	Zhou et al., 2014
28	**Potri.009G061500_MYB83**	20	Zhong et al., 2012	28	Potri.003G167900_TCP20	14	-	28	Potri.006G152700	10	-
29	Potri.010G093000_FLP	20	-	29	Potri.002G164400_WRKY22	13	-	29	Potri.018G068700	10	-
30	Potri.003G167900_TCP20	20	-	30	Potri.012G031700_WRKY53	13	-	30	**Potri.017G016700_SND2**	10	Zhong et al., 2008
31	Potri.003G142100_ARF9	19	-	31	Potri.002G180800_LHY	13	-	31	Potri.002G141200_At2g44940	10	-
32	Potri.012G031700_WRKY53	18	-	32	Potri.014G051200_At2g4473	12	-	32	Potri.017G082000_At1g26870	10	-
33	Potri.011G132400	18	-	33	Potri.001G088600_ARF9	12	-	33	Potri.004G159300_At2g16400	10	-
34	Potri.006G105300_WRKY33	18	-	34	Potri.001G154200_ERF6	12	-	34	Potri.010G099100_At2g02070	10	-
35	Potri.004G007500_WRKY42	18	-	35	Potri.017G068748_TCP20	12	-	35	**Potri.007G135300_SND2**	10	Zhong et al., 2008
36	Potri.016G128300_WRKY33	17	-	36	Potri.016G083600_TTG2	12	-	36	Potri.017G119900_C3H14	10	Chai et al., 2015
37	**Potri.013G113100_VND7**	17	Yamaguchi et al., 2011	37	Potri.004G007500_WRKY42	12	-	37	Potri.012G126500	10	-
38	Potri.014G100100_ARF9	17	-	38	Potri.006G133200_TTG2	12	-	38	Potri.001G137600_KAN2	10	-
39	Potri.011G007800_WRKY42	16	-	39	Potri.001G092900_WRKY53	12	-	39	Potri.002G181600_At3g61970	10	-
40	Potri.008G113200	16	-	40	**Potri.019G083600_VND7**	12	Yamaguchi et al., 2011	40	Potri.014G066100_At3g60580	10	-
41	**Potri.017G016700_SND2**	16	Zhong et al., 2008	41	Potri.010G006800	12	-	41	Potri.017G139500	10	-
42	Potri.006G221800_TT2	15	-	42	Potri.011G132400	11	-	42	Potri.008G106700	10	-
43	**Potri.019G083600_VND7**	15	Yamaguchi et al., 2011	43	Potri.009G055700_AGL15	11	-	43	Potri.002G154700_ANL2	10	-
44	Potri.001G080900_At2g43000	14	-	44	**Potri.013G113100_VND7**	11	Yamaguchi et al., 2011	44	Potri.007G014400	10	-
45	Potri.015G075600_GL1	14	-	45	Potri.002G055400_bHLH65	10	-	45	Potri.001G112200_KNAT7	10	Qin et al., 2020
46	Potri.002G172101_bHLH13	14	-	46	Potri.003G151000_At5g6159	9	-	46	Potri.002G034600	10	-
47	Potri.014G066100_At3g60580	14	-	47	Potri.001G079600_At5g6159	9	-	47	Potri.004G095100_C3H14	9	Chai et al., 2015
48	Potri.001G154200_ERF6	14	-	48	Potri.003G142100_ARF9	9	-	48	Potri.017G137600_KAN	9	-
49	**Potri.007G135300_SND2**	14	Zhong et al., 2008	49	Potri.006G005500_At5g4764	9	-	49	Potri.005G192000_At1g76890	9	-
50	Potri.003G080600_ERF6	13	-	50	Potri.012G138900_HSF6	9	-	50	Potri.014G080900	9	-
