# Supplementary material for: Gene regulatory network prediction using machine learning, deep learning, and hybrid approaches
Source: For Res (Fayettev). 2025 Jul 30;5:e014. doi: 10.48130/forres-0025-0014 (PMC12441907; doi:10.48130/forres-0025-0014)
Supplement: Supplementary file 1 — Supplementary data to this article can be found online. [file FR-2025-5-0014-Supplementary.zip › 10.48130_forres-0025-0014-Suppl-TableS3.pdf]

Suplementary Table S3. Comparison of the top 50 transcription factors (TFs) predicted to regulate the lignin biosynthesis pathway by hybrid Random Forest and plain Random Forest Models as well as a baseline method, Spearman's rank correlation on *Arabidopsis* Transcriptomic Test Data Set 1. The top 1000 predicted regulatory relationships was used to calculate the frequency of how many BLP genes each TF regulated. TFs highlighted in red represent known true regulators according to current literature, with the corresponding references provided.

Hybrid AdaBoost Model				Plain AdaBoost Model				Spearman Correlation Coefficient			
Rank	Transcription Factor	Freq.	Reference	Ran	Transcription Factor	Freq.	Reference	Rank	Transcription Factor	Freq.	Reference
1	**Zm00001eb076470_VND7**	34	Yamaguchi et al., 2011	1	Zm00001eb355240_AP2	34	-	1	Zm00001eb112680_bHLH62	13	-
2	**Zm00001eb176840_VND7**	34	Yamaguchi et al., 2011	2	Zm00001eb068520_AP2	34	-	2	Zm00001eb055830_FIT1	12	-
3	**Zm00001eb260000_VND7**	34	Yamaguchi et al., 2011	3	Zm00001eb265610_AP2	34	-	3	Zm00001eb426670_MYB15	12	
4	**Zm00001eb093920_MYB4**	34	Zhong et al., 2012	4	Zm00001eb062460_AP2	34	-	4	Zm00001eb077700_MYB15	12	-
5	Zm00001eb355240_AP2	34	-	5	Zm00001eb387280_AP2	34	-	5	Zm00001eb191940_GATA-12	11	-
6	Zm00001eb400120_SEP3	34	-	6	Zm00001eb050790_bHLH15	34	-	6	Zm00001eb164490	11	-
7	Zm00001eb070520_bHLH1	34	-	7	Zm00001eb122740_HY5	34	-	7	Zm00001eb318060	11	-
8	Zm00001eb311960_KAN	34	-	8	Zm00001eb231360_bZIP44	34	-	8	Zm00001eb159340_WRKY28	10	-
9	Zm00001eb102450_SEP3	34	-	9	Zm00001eb070520_bHLH15	34	-	9	Zm00001eb137920_WLIM1	10	-
10	Zm00001eb036590_SEP3	34	-	10	Zm00001eb432100_AP2	33	-	10	Zm00001eb320470_At3g5439	10	-
11	Zm00001eb317770_SEP3	34	-	11	Zm00001eb336820_HY5	32	-	11	Zm00001eb015120_MYB112	10	-
12	Zm00001eb122740_HY5	34	-	12	Zm00001eb235510_HY5	32	-	12	Zm00001eb044660_At5g6020	10	-
13	Zm00001eb424050_HY5	34	-	13	Zm00001eb417610_PIF3	31	-	13	Zm00001eb154170_WRKY69	10	-
14	Zm00001eb424040_HY5	34	-	14	Zm00001eb424050_HY5	31	-	14	Zm00001eb350280_WRKY33	9	-
15	Zm00001eb050790_bHLH1	34	-	15	Zm00001eb385610_HY5	31	-	15	Zm00001eb388620_WRKY40	9	-
16	Zm00001eb432100_AP2	34	-	16	Zm00001eb102450_SEP3	29	-	16	Zm00001eb294560_MYB86	9	-
17	Zm00001eb385610_HY5	34	-	17	Zm00001eb175540_bHLH137	29	-	17	Zm00001eb429870_ERF1	9	-
18	Zm00001eb068520_AP2	34	-	18	Zm00001eb072360	29	-	18	Zm00001eb393460_At5g0434	9	-
19	Zm00001eb265610_AP2	34	-	19	Zm00001eb189510_OBP4	29	-	19	Zm00001eb395580_MYB112	9	-
20	Zm00001eb387280_AP2	34	-	20	Zm00001eb424040_HY5	29	-	20	Zm00001eb223590_At1g7495	9	-
21	Zm00001eb336820_HY5	34	-	21	Zm00001eb391230_COL4	29	-	21	Zm00001eb327450_At1g7495	9	-
22	Zm00001eb062460_AP2	34	-	22	Zm00001eb338060_AG	29	-	22	Zm00001eb195420_WRKY55	9	-
23	Zm00001eb004600_bHLH1	34	-	23	Zm00001eb152350	27	-	23	Zm00001eb213800	9	-
24	Zm00001eb235510_HY5	34	-	24	Zm00001eb194220_At1g7808	27	-	24	Zm00001eb286490_WRKY33	9	-
25	Zm00001eb213550_bHLH9	33	-	25	Zm00001eb023220_COL4	25	-	25	Zm00001eb273610	9	-
26	Zm00001eb284010_AG	33	-	26	Zm00001eb427650_HB-7	24	-	26	Zm00001eb403720_KNAT7	9	Qin et al., 2020
27	Zm00001eb098330_WRKY	33	-	27	Zm00001eb004600_bHLH15	24	-	27	Zm00001eb169340_bHLH62	9	-
28	Zm00001eb338060_AG	33	-	28	Zm00001eb400130_CRC	24	-	28	Zm00001eb155610	9	-
29	**Zm00001eb410950_MYB4**	32	Zhong et al., 2012	29	Zm00001eb327040_AP1	24	-	29	Zm00001eb001720_KNAT7	9	Qin et al., 2020
30	Zm00001eb327040_AP1	31	-	30	Zm00001eb335690_At2g3809	24	-	30	Zm00001eb006180	8	-
31	Zm00001eb118120_AP1	30	-	31	Zm00001eb284010_AG	24	-	31	Zm00001eb153330_WRKY	8	-
32	Zm00001eb172450_LHY	30	-	32	Zm00001eb008690_AGL4	24	-	32	Zm00001eb074930_ERF1	8	-
33	Zm00001eb359470_WRKY	30	-	33	Zm00001eb311960_KAN	24	-	33	Zm00001eb330910	8	-
34	Zm00001eb145460_AG	29	-	34	Zm00001eb317770_SEP3	24	-	34	Zm00001eb072200	8	-
35	Zm00001eb310270_WRKY	28	-	35	Zm00001eb006180	19	-	35	**Zm00001eb157260_SND2**	8	Zhong et al., 2008
36	Zm00001eb406030_WRKY	28	-	36	Zm00001eb036590_SEP3	19	-	36	Zm00001eb125240_At1g6836	8	-
37	Zm00001eb344810_WRKY	28	-	37	Zm00001eb400120_SEP3	18	-	37	**Zm00001eb260850_NST2**	8	Mitsuda et al., 2007
38	Zm00001eb376400_WRKY	27	-	38	Zm00001eb348560	18	-	38	Zm00001eb185160_MYB15	8	-
39	Zm00001eb159410_WRKY	27	-	39	**Zm00001eb076470_VND7**	18	Yamaguchi et al., 2011	39	Zm00001eb342580_MYB55	8	-
40	Zm00001eb184340	27	-	40	Zm00001eb209070_bZIP11	16	-	40	**Zm00001eb269810_NST2**	8	Mitsuda et al., 2007
41	Zm00001eb120710_AG	26	-	41	Zm00001eb327140_NF-YB3	16	-	41	Zm00001eb417490_WRKY42	8	-
42	Zm00001eb358680_WRKY	26	-	42	Zm00001eb028820_MYB59	16	-	42	Zm00001eb335320_At1g6836	8	-
43	Zm00001eb041650_AGL15	25	-	43	Zm00001eb138380_SHP2	15	-	43	Zm00001eb068530	8	-
44	Zm00001eb344160_WRKY	24	-	44	Zm00001eb051380_At5g3966	15	-	44	Zm00001eb210520	7	-
45	Zm00001eb203940_WRKY	24	-	45	Zm00001eb144340_GAI	15	-	45	Zm00001eb326170_At3g4993	7	-
46	Zm00001eb134890_ZFP7	22	-	46	Zm00001eb066100_At5g6694	15	-	46	Zm00001eb030190_ZAT10	7	-
47	Zm00001eb193550_At1g21	20	-	47	Zm00001eb357220_At3g1381	12	-	47	Zm00001eb290350_WRKY33	7	-
48	Zm00001eb202570	20	-	48	Zm00001eb310270_WRKY53	11	-	48	Zm00001eb195770	7	-
49	Zm00001eb312620_hb-30	19	-	49	Zm00001eb139600_MYB73	11		49	Zm00001eb154560_MYB87	7	-
50	Zm00001eb348560	19	-	50	Zm00001eb051900_At4g3461	11	-	50	Zm00001eb326170_At3g4993	7	-
